# Deubiquitinase USP9X deubiquitinates β-catenin and promotes high grade glioma cell growth

**DOI:** 10.18632/oncotarget.12819

**Published:** 2016-10-22

**Authors:** Bo Yang, Shiming Zhang, Zhihao Wang, Chunxu Yang, Wen Ouyang, Fuxiang Zhou, Yunfeng Zhou, Conghua Xie

**Affiliations:** ^1^ Department of Radiation and Medical Oncology, Zhongnan Hospital, Wuhan University, Wuchang District, Wuhan, 430071, China; ^2^ Hubei Key Laboratory of Tumor Biological Behaviors, Zhongnan Hospital, Wuhan University, Wuchang District, Wuhan, 430071, China; ^3^ Department of Oncology, Wuhan General Hospital of Guangzhou Command PLA, Wuchang District, Wuhan, 430070, China

**Keywords:** high grade gliomas, USP9X, β-catenin, deubiquitination, prognosis

## Abstract

β-catenin is a crucial signal transduction molecule in the Wnt/β-catenin signal pathway, and increased β-catenin expression has consistently been found in high grade gliomas. However, the mechanisms responsible for β-catenin overexpression have remained elusive.

Here we show that the deubiquitinase USP9X stabilizes β-catenin and thereby promotes high grade glioma cell growth. USP9X binds β-catenin and removes the Lys 48-linked polyubiquitin chains that normally mark β-catenin for proteasomal degradation. Increased USP9X expression correlates with increased β-catenin protein in high grade glioma tissues. Moreover, patients with high grade glioma overexpressing USP9X have a poor prognosis. Knockdown of USP9X suppresses cell proliferation, inhibits G1/S phase conversion, and induces apoptosis in U251 and A172 cells. Interestingly, c-Myc and cyclinD1, which are important downstream target genes in the Wnt/β-catenin signal pathway, also show decreased expression in cells with siRNA-mediated down-regulation of USP9X. Down-regulation of USP9X also consistently inhibits the tumorigenicity of primary glioma cells in vivo.

In summary, these results indicate that USP9X stabilizes β-catenin and activates Wnt/β-catenin signal pathway to promote glioma cell proliferation and survival. USP9X could also potentially be a novel therapeutic target for high grade gliomas.

## INTRODUCTION

Gliomas account for the majority of all primary brain and central nervous system tumors in adults [1, 2]. High grade gliomas (WHO grade III–IV) are malignant and carry a worse prognosis even after standard treatment with surgery, ionizing radiation, and temozolomide [3, 4]. As they progress rapidly and are resistant to therapy, the median survival time of patients with glioblastoma multiforme (WHO grade IV) is approximately one year, and two years for anaplastic astrocytoma (WHO grade III) [5]. Thus, searching for a novel and specific therapeutic target is very important to improve the outcome of patients with high grade gliomas.

The Wnt/β-catenin signal pathway plays a key role in development, tissue homeostasis and cancer [6–8]. β-catenin is a crucial signal transduction molecule in the Wnt/β-catenin signal pathway [9–11]. Increased β-catenin expression has consistently been found in astrocytic tumors, in contrast to normal brain regions, and has been correlated with poor prognosis and short survival in glioblastoma multiforme patients [12, 13]. However, the mechanisms of β-catenin overexpression in glioma cells remain elusive. In the cytoplasm, β-catenin is ubiquitinated and degraded by the ubiquitin–proteasome pathway [14, 15]. The ubiquitination of substrates is a reversible reaction, and the ubiquitinated substrates are deubiquitinated by so-called deubiquitinating enzymes [16]. USP9X has been shown to interact with β-catenin both in vivo and in vitro in mouse lymphoma cells. The USP9X -binding site of β-catenin has been mapped to a region close to the adenomatous polyposis coli (APC) or Axin-binding site of β-catenin [17]. Overexpression of USP9X results in an elevation of β-catenin levels and an elongation of the half-life of β-catenin.

Here, we hypothesize that USP9X interacts with and stabilizes β-catenin in glioma cells. As a result, the Wnt/β-catenin signal pathway is activated and promotes glioma cell proliferation.

## RESULTS

### Increased USP9X expression correlated with poor prognosis in high grade gliomas

**Figure 1:**
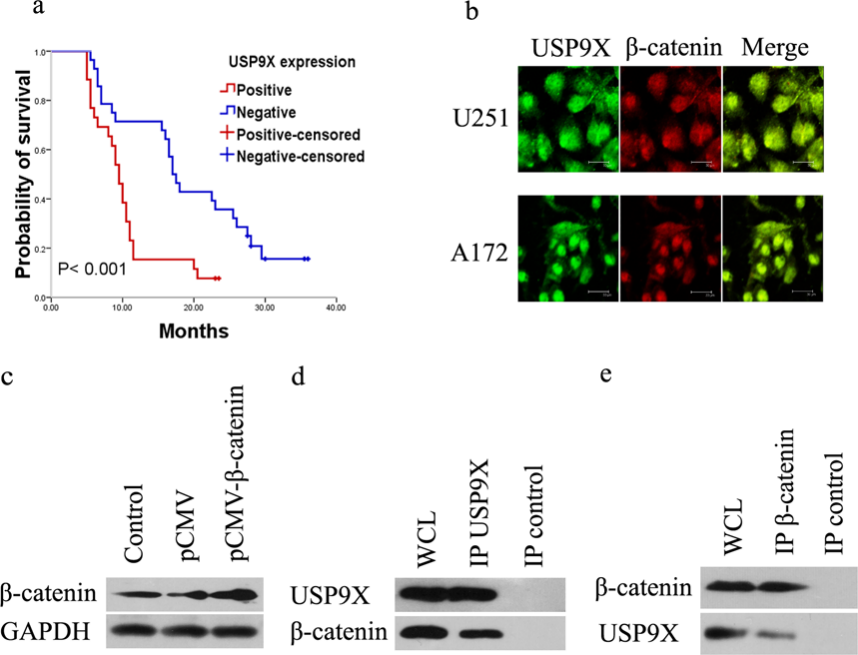
USP9X interacted with β-catenin **a.** Kaplan–Meier curves of high grade glioma patients with negative- versus positive-expression of USP9X in 54 high grade glioma patients. **b.** Co-localization of USP9X and β-catenin in U251 and A172 cells was detected by immunofluorescence. **c.** Western blot analysis of vector-induced overexpression of β-catenin in HEK293T cells after treatment with pCMV-β-catenin plasmid. **d** and **e.** HEK293T cells treated with pCMV-β-catenin plasmid for 48 h were used for endogenous USP9X and β-catenin immunoprecipitation. WCL, whole cell lysate.

The clinical materials of 54 high grade glioma patients were collected and tumor tissues from these patients were immunohistochemically assessed to detect USP9X expression. When comparing USP9X status with clinicopathological variables, we found no significant positive correlations between USP9X expression and age, gender, KPS, histology, tumor size, surgery, radiotherapy and chemotherapy (Table 1). Kaplan-Meier analysis showed that the differences in the survival of the USP9X expression group was highly statistically significant (Log-rank test, 10.618, *P*= 0.001) (Figure 1a). Meanwhile, the differences in the survival of the radiotherapy group and tumor size group were also statistically significant (Log-rank test, 4.548, *P* = 0.033; Log-rank test, 3.862, *P* = 0.049). Importantly, there were no significant differences between the two groups in terms of patient age, gender, KPS, histologic grade, surgery and chemotherapy (Supplementary Table S1). USP9X expression was a significantly independent prognostic factor (*P* = 0.002) with a relative risk of 0.365 (95% confidence interval, 0.193 – 0.688) in a Cox multivariate analysis, which was performed with the following variables for each case: USP9X expression, radiotherapy and tumor size (Supplementary Table S2). These results suggested that USP9X was indeed an independent negative prognostic factor for patients with high grade glioma and that USP9X-targeted studies could help explore new therapeutic strategies for this tumor.

**Table 1:** The correlation of USP9X expression and clinicopathological features in high grade glioma patients

Variable	Patients	USP9X		
		Positive cases	Negative cases	*P*
Age(years)				
Median	54	59	53	0.859
Range	16-67	16-67	30-61	
Gender				
male	34	17	17	0.783
Female	20	9	11	
KPS				
<90	22	9	13	0.418
≥90	32	17	15	
Histologic grade				
Grade 3	28	14	14	0.793
Grade 4	26	12	14	
Tumor size(cm)				
Mean	4.4	4	4.7	0.097
Range	2.5-8	2.5-6	3-8	
Surgery				
Subtotal	40	20	20	0.760
Near gross total resection	14	6	8	
Radiotherapy				
Yes	40	16	24	0.063
No	14	10	4	
Chemotherapy				
Yes	18	8	10	0.777
No	36	18	18	
Current status				
Alive	7	2	5	0.423
Died	47	24	23	

*Statistically significant.

### USP9X inhibition retarded WNT/β-catenin signal pathway through β-catenin

To further explore the molecular mechanisms of USP9X in high grade gliomas, we studied the key molecular pathways involved in action. The co-localization of USP9X and β-catenin was detected by using laser confocal microscopy. As shown in Figure 1b, both the cytoplasm and cell nuclei of the U251 cells and A172 cells were positively stained for USP9X and β-catenin. The merged graph suggested widespread co-localization in the U251 and A172 cells. The plasmid pCMV-β-catenin was constructed and transfected into HEK293T cells to overexpress β-catenin (Figure 1c). To identify whether the deubiquitinases USP9X interacted with β-catenin, we analyzed proteins by specifically co-immunoprecipitating specifically with USP9X and β-catenin antibodies from the HEK293T cells. The results of western blot analysis suggested that USP9X interacted specifically with β-catenin (Figure 1d) and that β-catenin also interacted specifically with USP9X (Figure 1e).

**Figure 2:**
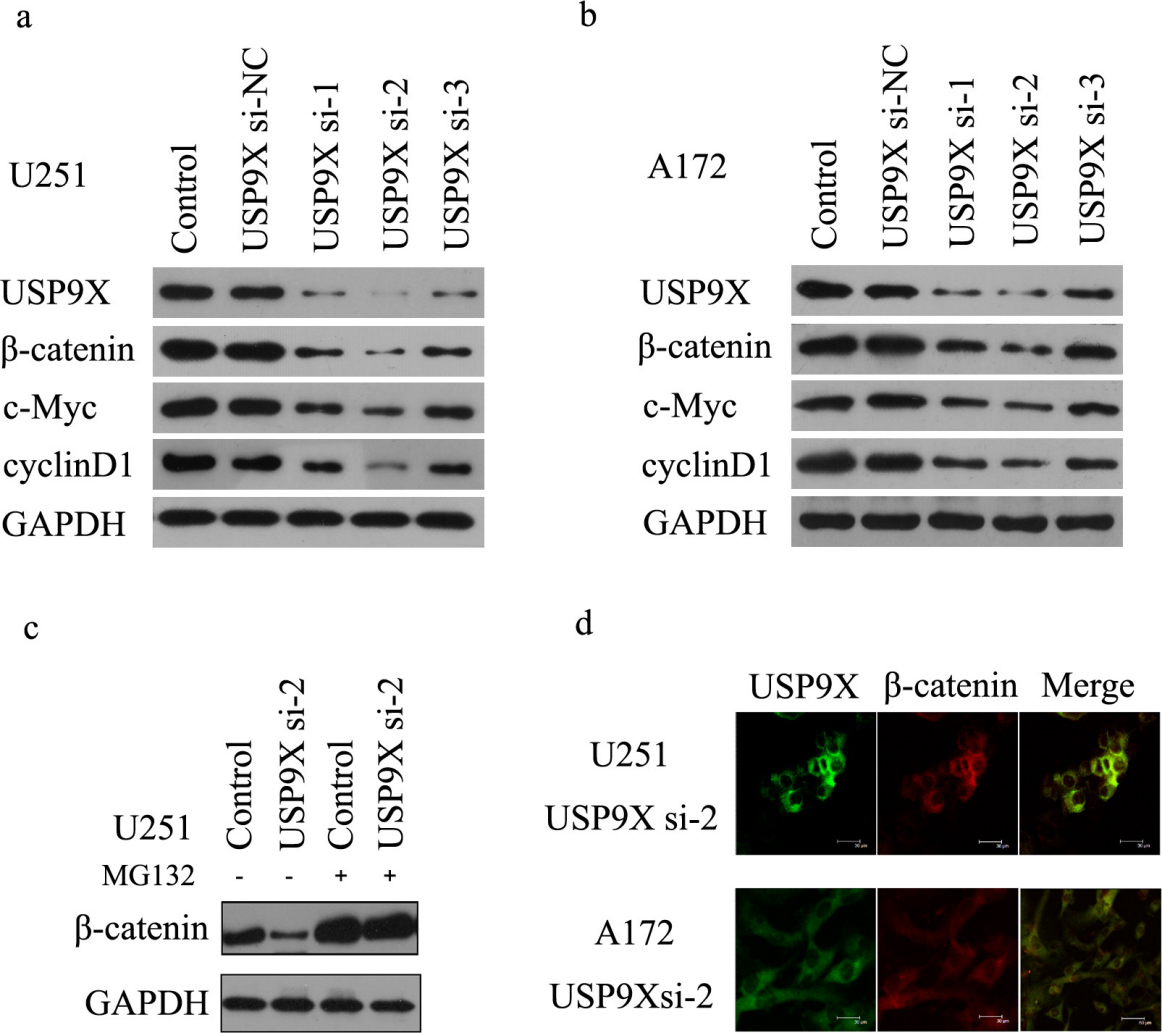
USP9X inhibition retarded WNT/β-catenin signal pathway **a** and **b.** Western blot analysis of USP9X, β-catenin. c-Myc and cyclinD1 levels after USP9X siRNAs were transfected into U251 and A172 cells, respectively for 72 h. **c.** MG-132 recovered β-catenin protein expression after USP9X knock down. USP9X si-2 was transfected into U251 cells for 72 hours and treated with or without 10mM MG-132 for 4 h, cells were harvested and lysed for western blot. **d.** Staining of β-catenin protein in U251 and A172 cells treated by USP9X si-2.

To obtain direct evidence that USP9X affected β-catenin stability, we transfected short interfering RNAs (siRNAs) targeting USP9X into U251 and A172 cells. The results of the western blot suggested that knockdown of USP9X decreased β-catenin protein (Figure 2a, 2b), but the results of RT-PCR suggested that β-catenin mRNA expression levels did not change (Supplementary Figure S1a, S1b). C-Myc and cyclinD1 are important downstream target proteins of the Wnt/β-catenin signaling pathway. The mRNA and protein expression of c-Myc and cyclinD1 all decreased (Figure 2a, 2b Supplementary Figure S1c-f) after transfection with USP9X siRNA. Interestingly, after MG-132 was added to inhibit the ubiquitin proteasomes pathway, the total amount of β-catenin protein immediately increased (Figure 2c). Next, we examined β-catenin by immunofluorescence after knockdown of USP9X. We found that β-catenin in U251 and A172 cell nuclei was remarkably decreased after the expression of USP9X protein was inhibited by siRNA-2 (Figure 2d).

**Figure 3:**
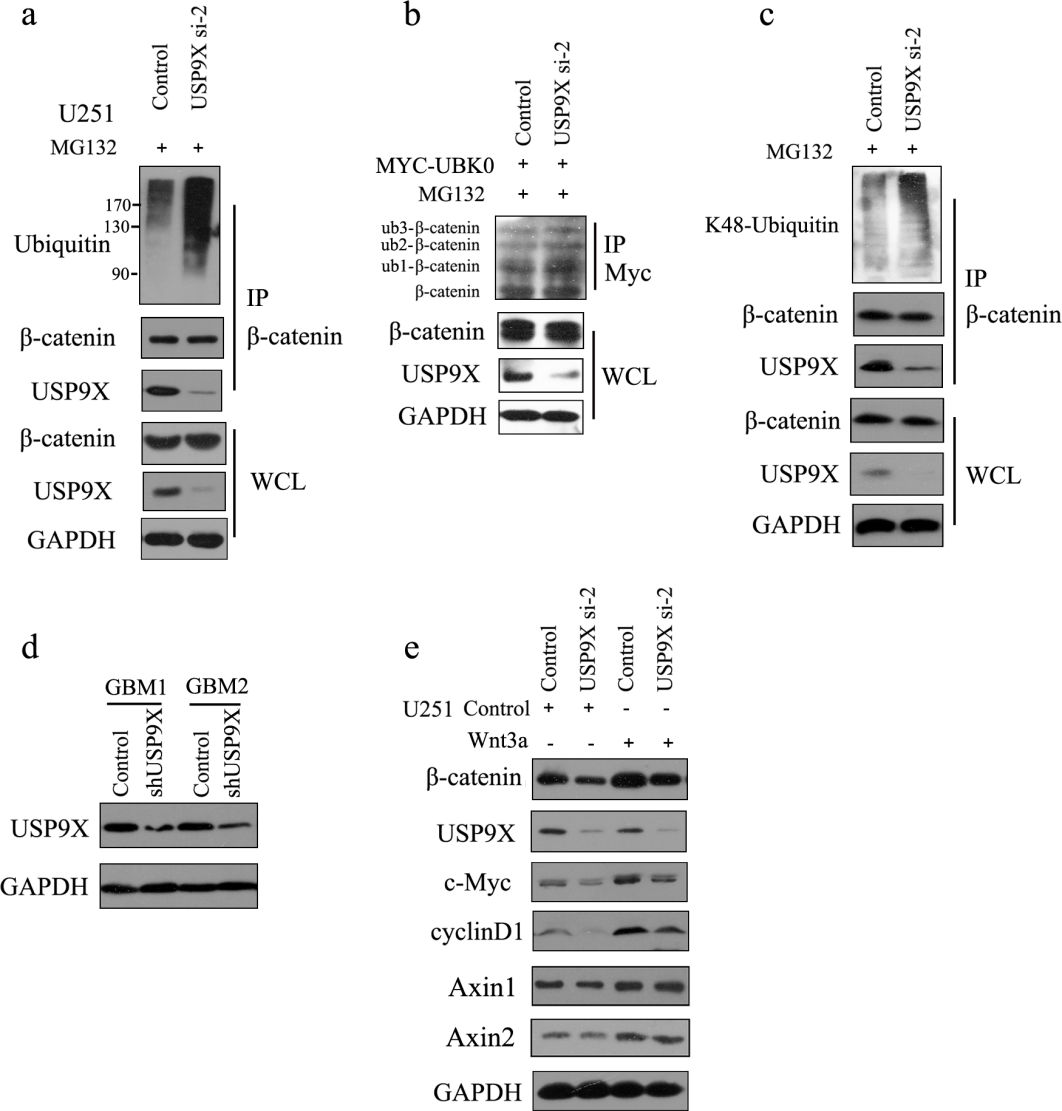
USP9X knockdown caused increase of K48-ubiquitinated β-catenin **a.** USP9X siRNA-2 was transfected into U251 cells, and after another 48 h, cells were treated with MG132 for 4 h before harvest. β-catenin was immunoprecipitated and immunoblotted with β-catenin antibodies or anti-ubiquitin antibodies. **b.** USP9X siRNA-2 was transfected into U251 cells along with Myc-UBK0 plasmid, and after another 48 h, cells were treated with MG132 for 4 h before harvest. Myc-UBK0 was immunoprecipitated and immunoblotted with β-catenin antibodies. **c.** USP9X siRNA-2 was transfected into U251 cells, and after another 48 h, cells were treated with MG132 for 4 h before harvest. β-catenin was immunoprecipitated and immunoblotted with K48-linkages specific ubiquitin antibody. **d.** The efficiency of USP9X shRNA was examined using anti-USP9X antibody. **e.** USP9X siRNA-2 was transfected into U251 cells, and after another 48 h, cells were stimulated with Wnt3a for 3 h and examined with indicated antibody.

**Figure 4:**
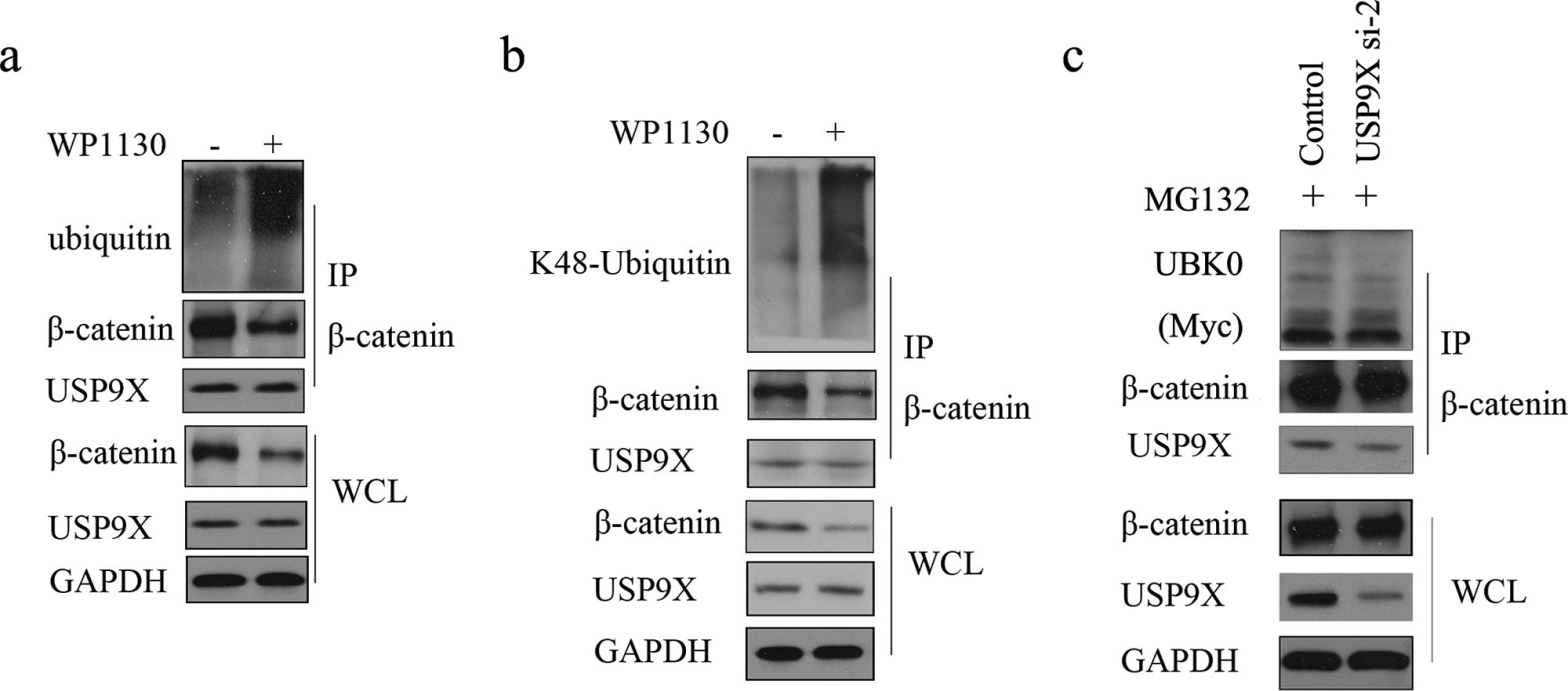
USP9X specific inhibitor WP1130 treatment increased of K48-ubiquitinated β-catenin **a.** U251 cells were treated with 1μM WP1130 for 24h before harvest. β-catenin was immunoprecipitated and immunoblotted with β-catenin antibody or anti-ubiquitin antibody. **b.** U251 cells were treated with 1μM WP1130 for 24h before harvest. β-catenin was immunoprecipitated and immunoblotted with β-catenin antibody or K48-linkages specific ubiquitin antibody. **c.** USP9X siRNA-2 was transfected into U251 cells along with Myc-UBK0 plasmid, and after another 48h, cells were treated with MG132 for 4 h before harvest. β-catenin was immunoprecipitated and immunoblotted with Myc-Tag antibody.

As shown in Figure 3a, knockdown of USP9X increased the ubiquitination of β-catenin and subsequent degradation. We also showed that USP9X did not affect the monoubiquitination of β-catenin(Figure 3b). Taken together, this indicated that USP9X stabilized β-catenin by increasing the ployubiquitination of β-catenin. K48-linked ubiquitination has been shown to be involved in protein degradation. By using a K48-linkage specific ubiquitin antibody, we showed that knockdown of USP9X could increase K48-linked ubiquitination of β-catenin (Figure 3c). To draw more accurate conclusions, here we used commercially available direct inhibitors of USP9X, WP1130 (from Selleck Chemicals, S2243) for the β-catenin ubiquitination assay. WP1130 acts as a partly selective deubiquitinase inhibitor, directly inhibiting deubiquitinase activity of USP9X, USP5, USP14, and UCH37, which are known to regulate survival protein stability and 26S proteasome function. Treatment of WP1130 clearly increased the ubiquitination of β-catenin in U251 cells (Figure 4a). By using a K48-linkage specific ubiquitin antibody, we showed that treatment of WP1130 could increase K48-linked ubiquitination of β-catenin (Figure 4b). USP9X siRNA-2 was transfected into U251 cells along with Myc-UBK0 plasmid, and the results also showed that USP9X did not affect the monoubiquitination of β-catenin (Figure 4c).

### Knockdown of USP9X inhibited cell proliferation and cell cycle progression and increased apoptosis

**Figure 5:**
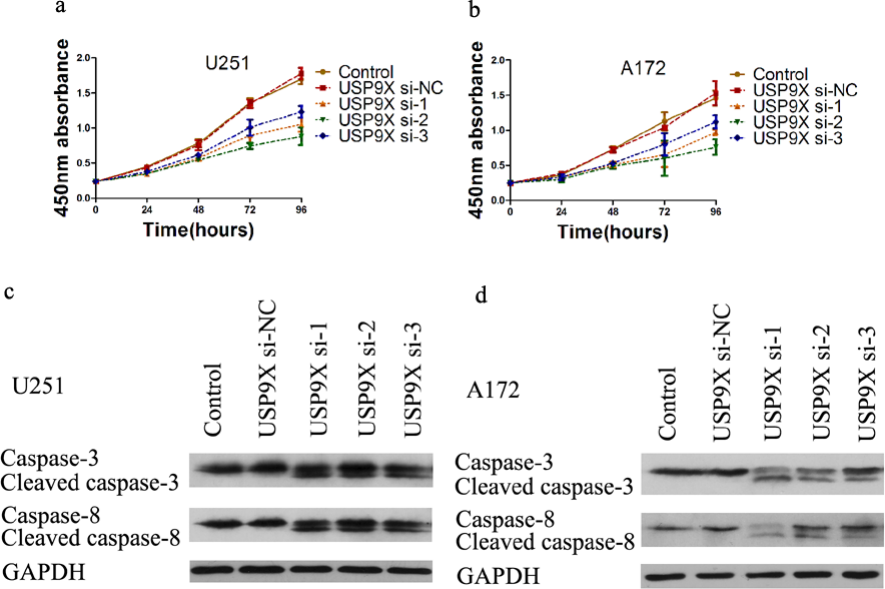
Cell proliferation decreased after USP9X knockdown **a** and **b.** Cell proliferation curves of U251 and A172 cells after USP9X knockdown. **c** and **d.** USP9X siRNAs were transfected into U251 and A172 cells for 72 h, cells were harvested and lysed for western blot to detect caspase 3 and caspase 8.

Compared with the control group and si-NC group, the absorbance at 450nm of the siRNA 1-3 groups was significantly lower than the two groups (*P* <0.05) (Figure 5a, 5b). We also explored the mechanism through which USP9X affected U251 and A172 cell proliferation abilities. After USP9X expression was suppressed by the siRNA, the expression of c-Myc mRNA and protein decreased. Subsequently, we detected whether USP9X affected the cell cycle of glioma cells. Compared with the control group and si-NC group, the G1 phase of the siRNA 1-3 groups was significantly longer (*P* <0.05) (Supplementary Figure S2a, S2b). In contrast, the duration of the S phase of the siRNA 1-3 groups decreased (*P* <0.05) (Supplementary Figure S2c,d). We also detected whether USP9X affected the apoptosis of glioma cells. Compared with the control group and si-NC group, the apoptosis rates of the siRNA 1-3 groups were significantly higher (*P* <0.05) (Supplementary Figure S2g, S2h). The results of the western blots suggested that cleaved caspase 3 and cleaved caspase 8 proteins emerged in the siRNA 1-3 groups (Figure 5c, 5d). However, there was no expression of cleaved caspase 3 and caspase 8 in the control group and si-NC group.

### The expression of USP9X was significantly correlated with β-catenin, c-Myc and cyclinD1 in high grade glioma tissues

**Figure 6:**
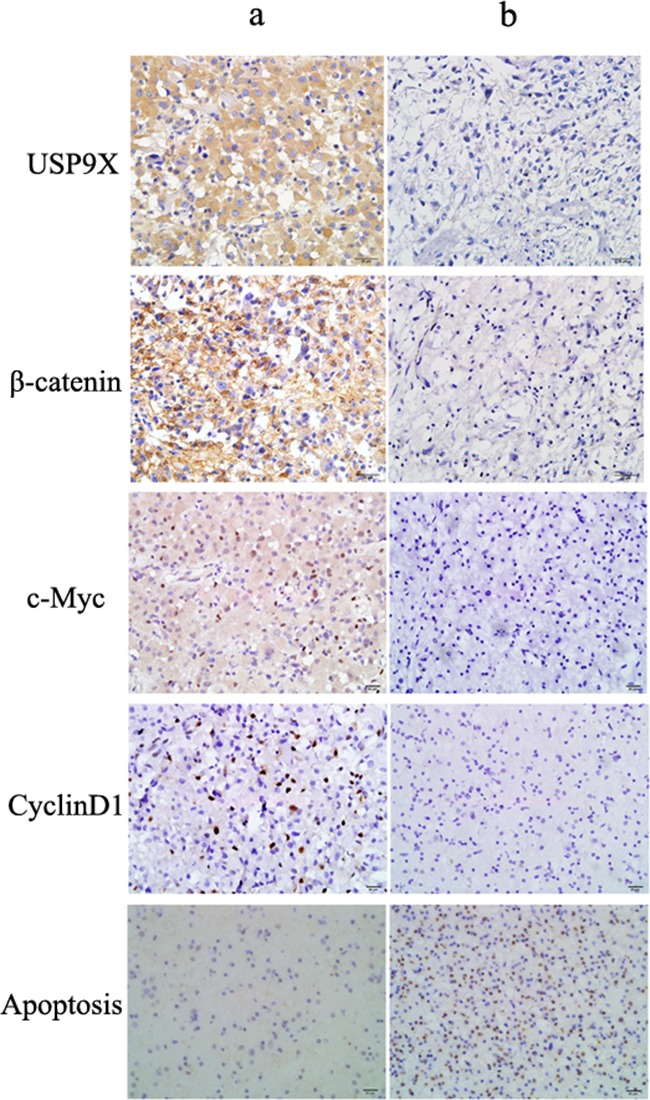
Representative immunohistochemical staining for USP9X, β-catenin, c-Myc and cyclinD1 in high grade glioma tissues **a.** Positive USP9X, β-catenin, c-Myc, cyclinD1 expression in patient a (Original magnification, ×400). **b.** Negative USP9X, β-catenin, c-Myc, cyclinD1 expression in patient b (Original magnification, ×400).

Among 54 glioma cases, there were 26 USP9X positive cases, representing a positive rate of 48.1% (Supplementary Table S3). Using immunohistochemistry, we found that USP9X positive reactions primarily occurred in the cytoplasm of high grade glioma cells and produced brownish yellow granules (Figure 6a). A total of 28 patients were β-catenin positive, representing a positive rate of 51.9%. β-catenin was primarily expressed in the cell membrane and cytoplasm of tumor cells, and a few cell nuclei were β-catenin positive. The expression of USP9X and β-catenin was significantly correlated (Spearman's rank test, *P* <0.001) in high grade glioma tissues. The positive rates of c-Myc and cyclinD1 were 48.1% and 40.7 %, respectively. We found that c-Myc and cyclinD1 positive reactions primarily occurred in the cell nuclei and produced brownish yellow granules (Figure 6a). The expression of USP9X was also significantly correlated with the expression of c-Myc and cyclinD1 in high grade glioma tissues (Spearman's rank test, *P* <0.001). Moreover, the rate of apoptosis in USP9X positive cases was 5.2 ± 0.8%, and the rate of apoptosis in USP9X negative cases was 28.7 ± 1.2%, as detected by TUNEL method. As shown in Figure 6a,b, apoptotic cells were rarely observed in USP9X positive patients (left) and quite a few apoptotic cells were observed in USP9X negative patients (right).

### Downregulation of USP9X decreased tumorigenesis of glioma cells in vivo

**Figure 7:**
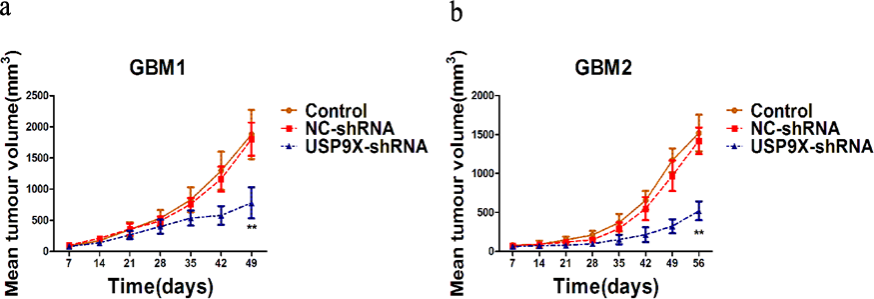
USP9X knockdown decreased tumorigenesis of glioma cells in vivo **a** and **b.** Growth of GBM1and GBM2 xenograft tumors expressing USP9X-shRNA or NC-shRNA. Control, GBM1 and GBM2 cells.

To determine the role of USP9X in modulating glioma tumorigenesis in vivo, we implanted primary glioma cells and lentivirus infected primary glioma cells into the dorsal side of the left hind legs of mice. The efficiency of USP9X shRNA was also examined using anti-USP9X antibody (Figure 3d). As shown in Figure 7, the USP9X-shRNA group formed much smaller tumors and displayed reduced tumorigenicity, showing that downregulation of USP9Xreduced primary glioma cell tumorigenesis in vivo. The NC-shRNA group formed tumors similar to the control group. We consistently demonstrated that USP9X affected U251 and A172 cell proliferation abilities. So, knockdown of USP9X not only inhibited the proliferation of glioma cells in vitro, but also suppressed the tumorigenicity of primary glioma cells in vivo.

## DISCUSSION

USP9X is a member of the peptidase C19 family. A few studies have explored the relationship between USP9X and tumors, but the results have been controversial. Immunohistochemical staining of follicular lymphomas and normal lymphoid tissue samples have shown that USP9X levels are increased in the lymphomas [18]. Increased USP9X mRNA in tumors has been significantly associated with poor prognosis for patients with multiple myeloma, and patients highly expressing USP9X mRNA have a 5.5-fold greater risk of death [19, 20]. In contrast, Pérez-Mancera PA, *et al*. found that low USP9X protein and mRNA expression in pancreatic ductal adenocarcinoma (PDA) correlated with poor survival after surgery and USP9X levels were inversely associated with metastatic burden in advanced disease [21]. These results suggested that USP9X might have different clinicopathological and prognostic significance in different tumors.

Next, we demonstrated that USP9X inhibition retarded the Wnt/β-catenin signal pathway through β-catenin. As transcriptional regulation of c-myc and cyclin D1 may be manifested indirectly by reduced cell cycle progression induced by USP9X knockdown, we need to clarify whether USP9X can regulate c-myc and cyclin D1 levels through the Wnt/β- catenin pathway. By evaluating signaling in U251 cells treated with a Wnt ligand, we found that USP9X knockdown partly blocked the activation of Wnt/β- catenin pathway by regulating the stability of β-catenin (Figure 3e). c-Myc promotes cell division and unlimited proliferation of glioma cells and glioma stem cells [22–24]. The overexpression of c-Myc oncogene is associated with pathogenesis of many cancers [25]. CyclinD1 is required for the G1/S transition in the cell cycle. Mutation, amplification and overexpression of cyclinD1, which alters cell cycle progression, are observed frequently in a variety of tumors and may contribute to tumorigenesis [26–28]. Previous studies have suggested that deubiquitinase USP9X stabilizes myeloid cell leukemia sequence 1 (MCL1) and promotes tumor cell survival and apoptosis resistance [29, 30]. In this study, our data indicated that USP9X could also affect the proliferation, cell cycle and apoptosis of U251 and A172 cells through regulating the Wnt/β-catenin signal pathway.

However, β-catenin plays an important role in the Wnt/β-catenin signal pathway. Knockdown of β-catenin in glioma cells inhibited cell proliferation and resulted in cell apoptosis [31, 32]. Previous studies have suggested that USP9X is co-immunoprecipitated with β-catenin and inhibits the degradation of β-catenin through the deubiquitination of β-catenin in mouse lymphocyte cells [17]. The results of our experiments suggested that USP9X affected β-catenin post-transcriptionally, and USP9X might regulate the amount and distribution of β-catenin protein. Failure to eliminate damaged cells by apoptosis is vital in tumor development [33]. Cleaved caspase 3 and caspase 8 are central players in programmed cell death [34, 35]. As inhibition of USP9X resulted in caspase 3 and caspase 8 activation and increased the rates of apoptosis, USP9X might promote cell survival by inhibiting apoptosis in glioma cells.

In summary, our study indicated that USP9X expression appeared as a significantly independent prognostic factor of high grade glioma patients, and USP9X interacted with and stabilized β-catenin and activated the WNT/β-catenin signal pathway to promote glioma cells proliferation and survival in vitro and in vivo. Our findings may provide new evidence that regulation of USP9X could potentially be a plausible method for improvement of prognosis in high grade glioma patients. USP9X could also potentially be a therapeutic target in high grade gliomas.

## MATERIALS AND METHODS

### Patient samples

A total of 54 paraffin-embedded, high-grade, WHO grading system III to IV glioma tumor samples were used in this study. They were obtained from the Department of Pathology, Wuhan General Hospital of Guangzhou Command. The samples were collected from patients undergoing surgery for high grade gliomas from 2004 to 2009. After resection of tumor, some patients received radiotherapy and chemotherapy, and the other patients who rejected postoperative treatment were treated with supportive care. This study was approved by the Ethics Committee of Wuhan General Hospital of Guangzhou Command.

### Immunohistochemistry

The paraffin embedded stage III to IV glioma tumor tissues were stained with a commercially available anti-USP9X antibody (0.1ug/ml; ab19879, Abcam Biotechnology) and anti-β-catenin antibody (0.1ug/ml; ab22656, Abcam Biotechnology). Immunohistochemistry stained slides were independently reviewed by two pathologists who were blinded to all clinical data. Staining was graded (0, negative; 1, weak; 2, moderate; 3, strong) and the percentage of positively stained cells was counted (0, ≤ 5%; 1, 6%-25%; 2, 26%-49%; 3,≥ 50%). The final score was determined by the combined staining score (intensity + extent). A score <3 was defined as negative expression, and ≥3as a positive staining pattern.

### USP9X silencing in glioma cells

U251and A172 human malignant glioma cell lines were obtained from the Cell Bank of Chinese Academy of Science, Shanghai, China. USP9X was knocked down by using 3 validated, commercially available USP9X-specific siRNAs (USP9X si-1, USP9X si-2 and USP9X si-3) and a nontarget, scrambled siRNA (USP9X si-NC) sequence as a negative control (Shanghai GenePharma Co.Ltd). The sequences were as follows: USP9X si-1(5′ CCCGCACUGAAACAAAUUATT 3 ', 5′ UAAUUUGUUUCAGUGCGGGTT 3′), USP9X si-2(5′ GACCUCAUCAGAUUAUUAUTT 3′,5′ AUA AUAAUCUGAUGAGGUCTT 3′), USP9X si-3(5′ GG GCAAUGGAGAUCUUAAATT 3′,5′ UUUAAGAUCU CCAUUGCCCTT 3′), USP9X si-NC (5′ UUCUCCGAAC GUGUCACGUTT 3′,5′ ACGUGACACGUUCGGAGA ATT 3′). Transfection was carried out using siRNA duplexes at a final concentration of 100nmol/L and Lipofectamine RNAiMAX reagent (Life Technologies).

### Cell proliferation

U251 and A172 glioma cells were seeded in 96-well plates (2000 cells/well). After serum starvation, the cells were further transfected with USP9X-specific siRNAs. The proliferative potential of cells was analyzed according to the protocol of CCK-8 (Dojindo, Japan). After 24h, 48h, 72h and 96h, the absorbance at 450 nm was measured using a microplate reader with the wavelength correction set to 630 nm.

### β-catenin overexpression and immunoprecipitation

The HEK293T cell line was obtained from the Cell Bank of Chinese Academy of Science, Shanghai, China. The full-length β-catenin gene was delivered into the HEK293T cells via the pCMV-β-catenin plasmid. Forty-eight hours after transduction, cells were harvested. The lysates were then resolved by SDS-PAGE and immunoblotted with indicated antibodies.

### Quantitative realtime RT-PCR, western blot analysis and FCM measurement

U251 and A172 cells were cultured in 40 mm dishes and further transfected with USP9X-specific siRNAs. After a 48 h culture, the total cell RNA in each dish was extracted and quantitative realtime RT-PCR was performed according to the protocol (TAKARA, Japan). Primer sequences are shown in data Supplementary Table S4. All samples were examined in triplicate and included no-template controls. After 72 h cultures, cells were lysed and western blotted. The cell cycles and apoptosis rates were detected by flow cytometry.

### Glioma primary cell cultures and lentivirus infections

Primary cell cultures (GBM1, GBM2) were established from glioblastoma multiforme tissues obtained during surgery and diagnosed according to the WHO classification. The protocol was approved by the Ethics Committee of Wuhan General Hospital, Guangzhou Command. Selected shRNA lentivirus vector (pGMLV-SC5) against USP9X (targeting sequence: 5′-CAATCAAGTTCAATGATTA-3′) and the negative control shRNA with GFP were obtained as a kit from Genomeditech Co., Ltd. (Shanghai, China). After lentivirus infection of GBM1and GBM2, GFP+ cells were sorted by flow cytometry and single cell colonies were grown.

### Glioma xenograft model

Animal experiments were performed in accordance with animal care ethics approval and guidelines. BALB/C nude mice aged 4 to 6 weeks were purchased from Shanghai Experimental Animal Center (Chinese Academy of Sciences, Shanghai, China). GBM1 and GBM2 cells (5×10^6^ in 100 μL media) were injected subcutaneously into the left dorsal leg of the mice. To follow tumor growth, tumor size was measured with a caliper each week, and tumor volume was calculated using the formula: volume = length × width^2^ × π/6. The endpoint of observation was at the end of the eighth week.

### Statistical analysis

SPSS software (version 17; IBM Corporation) was used to conduct all statistical tests. All in vitro experiments were repeated independently in triplicate. Data were pooled with mean and SE of the mean values calculated for all end points where appropriate. The 2-tailed Student t test was used to test the significance of differences in sample means for data with normally distributed means. Alternatively, the Mann–Whitney U test was used for the analysis of nonparametric data. Kaplan–Meier survival curves were generated and compared using a 2-sided log-rank statistic. The Cox proportional hazards model was used for the multivariate analysis of patient survival. The Pearson correlation coefficient was used to test linear associations. A *P* value less than 0.05 (two-sided) was considered significant.

## SUPPLEMENTARY FIGURES AND TABLES


