# Supplementary material for: Deubiquitinase USP9X deubiquitinates β-catenin and promotes high grade glioma cell growth
Source: Oncotarget. 2016 Oct 22;7(48):79515–25. doi: 10.18632/oncotarget.12819 (PMC5346732; doi:10.18632/oncotarget.12819)
Supplement: Supplementary file 1 [file oncotarget-07-79515-s001.pdf]

Deubiquitinase USP9X deubiquitinates β-catenin and promotes high grade glioma cell growth

SUPPLEMENTARY FIGURES AND TABLES

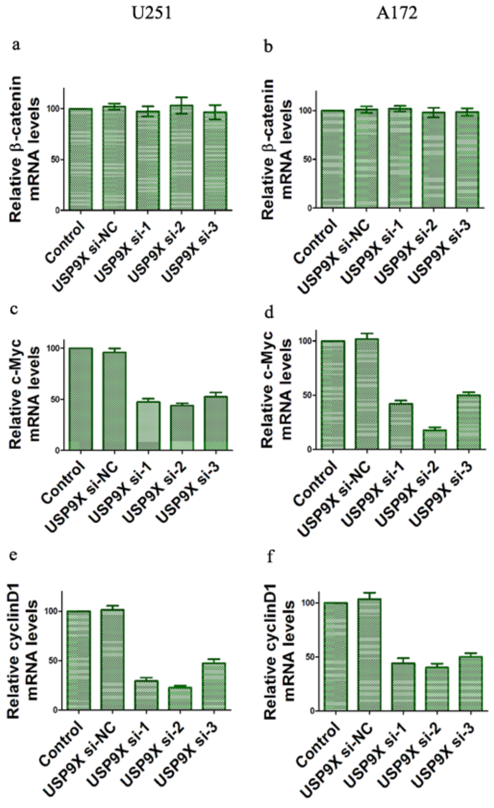

Supplementary Figure S1: USP9X inhibition regulated β-catenin, c-Myc and cyclinD1 mRNA levels. a-f. RT-PCR analysis of β-catenin. c-Myc and cyclinD1 mRNA levels after USP9X siRNAs were transfected into U251 and A172 cells respectively for 48 h.

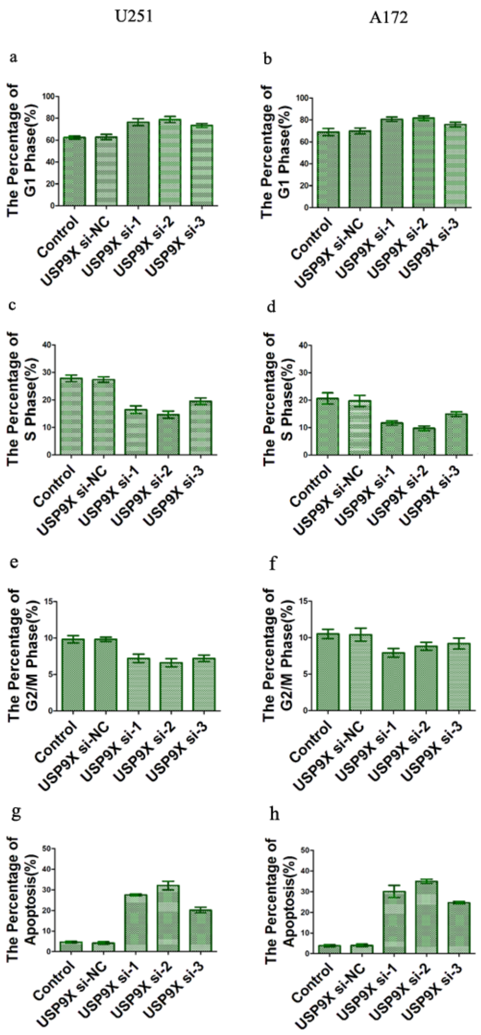

Supplementary Figure S2: Cell cycle retardation and apoptosis increased after USP9X knockdown. a-f. The percentages of G1, S and G2/M phase of U251 and A172 cells transfected with siRNAs targeting USP9X. g and h: The apoptosis rates of U251 and A172 cells after USP9X knockdown.

Supplementary Table S1: Results of survival analysis for individual factors

Factor	Log rank	*P*
Age	2.240	0.135
Gender	0.228	0.633
KPS	0.038	0.846
Histologic grade	0.034	0.854
Tumor size	3.862	0.049
surgery	0.142	0.706*
Radiotherapy	4.548	0.033*
Chemotherapy	0.378	0.539
USP9X expression	10.618	0.001*

*Statistically significant.

Supplementary Table S2: Results of Cox multivariate regression factors

Factor	Wald value	*P* value	95.0% CI
Tumor size	0.133	0.715	0.614-2.035
Radiotherapy	0.933	0.334	0.364-1.410
USP9X expression	9.714	0.002*	0.193-0.688

*Statistically significant.

Supplementary Table S3: The expression of USP9X, β-catenin, c-Myc and cyclinD1 in high grade glioma patients

USP9X	β-catenin		c-myc		cyclinD1	
	negative	positive	negative	positive	negative	positive
negative	24	4	24	4	26	2
positive	2	24	4	22	6	20

Supplementary Table S4: PCR Primer sequences of USP9X, β-catenin, c-Myc, cyclinD1 and GAPDH

Primers	Forward primer sequence (5'-3')	Reverse primer sequence (5'-3')
USP9X	GCCGAAAGGGGAATTAGAAG	ACTTCCAGCCACTCACTGCT
β-catenin	GAAACGGCTTTCAGTTGAGC	CTGGCCATATCCACCAGAGT
c-myc	GGGTAGTGGAAAACCAGCAGC	CCTCCTCGTCGCAGTAGAAATA
Cyclin D1	GAGGAACAGAAGTGCGAGGAG	GGATGGAGTTGTCGGTGTAGAT
GAPDH	TGGAAGGACTCATGACCACA	TTCAGCTCAGGGATGACCTT
